# Improvement of Heat Resistance of Fluorosilicone Rubber Employing Vinyl-Functionalized POSS as a Chemical Crosslinking Agent

**DOI:** 10.3390/polym15051300

**Published:** 2023-03-04

**Authors:** Jae Il So, Chung Soo Lee, Byeong Seok Kim, Hyeon Woo Jeong, Jin Sung Seo, Sung Hyeon Baeck, Sang Eun Shim, Yingjie Qian

**Affiliations:** Department of Chemistry and Chemical Engineering, Education and Research Center for Smart Energy and Materials, Inha University, Incheon 22212, Republic of Korea

**Keywords:** fluorosilicone, polyhedral oligomeric silsesquioxane, chemical crosslinking agent, heat resistance

## Abstract

Fluorosilicone rubber (F-LSR) is a promising material that can be applied in various cutting-edge industries. However, the slightly lower thermal resistance of F-LSR compared with that of conventional PDMS is difficult to overcome by applying nonreactive conventional fillers that readily agglomerate owing to their incompatible structure. Polyhedral oligomeric silsesquioxane with vinyl groups (POSS-V) is a suitable material that may satisfy this requirement. Herein, F-LSR-POSS was prepared using POSS-V as a chemical crosslinking agent chemically bonded with F-LSR through hydrosilylation. All F-LSR-POSSs were successfully prepared and most of the POSS-Vs were uniformly dispersed in the F-LSR-POSSs, as confirmed by Fourier transform infrared spectroscopy (FT-IR), proton nuclear magnetic resonance spectroscopy (^1^H-NMR), scanning electron microscopy (SEM), and X-ray diffraction (XRD) measurements. The mechanical strength and crosslinking density of the F-LSR-POSSs were determined using a universal testing machine (UTM) and dynamic mechanical analysis (DMA), respectively. Finally, differential scanning calorimetry (DSC) and thermogravimetric analysis (TGA) measurements confirmed that the low-temperature thermal properties were maintained, and the heat resistance was significantly improved compared with conventional F-LSR. Eventually, the poor heat resistance of the F-LSR was overcome with three-dimensional high-density crosslinking by introducing POSS-V as a chemical crosslinking agent, thereby expanding the potential fluorosilicone applications.

## 1. Introduction

Fluorosilicone is a promising material owing to its excellent oil resistance, chemical resistance, and low-temperature elasticity retention compared with general PDMS [1,2,3,4]. However, the high-temperature application of fluorosilicone is limited because its stability is somewhat inferior to that of conventional silicone at high temperatures, owing to the homolytic scission of trifluoropropyl groups [5]. To compensate for these poor properties, fillers, including silica, titania, zirconia, Al_2_O_3_, carbon black, and carbon nanotubes, have been applied to fluorosilicone [6,7,8,9,10,11,12,13,14]. Silica is a widely used filler because it is composed of Si and O, sharing the same atoms as silicone polymers.

A large amount of silica can be introduced into silicone matrices compared to other fillers owing to the similar compositions, without significantly changing the original properties and color of silicone. Nevertheless, the agglomeration of silica particles can occur inside the silicone matrix, thus resulting in some silicone property degradation [6,15]. To prevent this agglomeration, the filler must be thoroughly dispersed in the silicone matrix and chemically bonded with silicone. Therefore, a material with a low molecular weight, a molecular structure similar to that of silicone, and numerous crosslinking sites with silicone is required. A material that satisfies all these requirements is octafunctional polyhedral oligomeric silsesquioxane (T-8 POSS).

T-8 POSS of the cage-type silsesquioxane with the general formula (RSiO_1.5_)_8_ comprises a robust framework and eight organic functional groups [16,17]. Silicone crosslinking involves bonding Si-vinyl in the main chain and Si–H in the crosslinker over platinum catalysts. T-8 POSS, with different numbers of vinyl groups, is a beneficial crosslinking agent because the Si-vinyl group in T-8 POSS can connect with the main chain and crosslinker via hydrosilylation [18,19]. Moreover, T-8 POSS can contain up to eight vinyl groups with low molecular weights, which improves the dispersion and crosslinking inside the silicone [20,21,22]. Accordingly, several studies have used octavinyl-POSS as a crosslinking agent in various polymers [23,24,25,26].

Previous studies have reported that the thermal properties are improved when octavinyl-POSS with eight vinyl groups is used as a crosslinking agent [26,27,28]. Using octavinyl-POSS as a filler can increase the polymer’s thermal properties because the POSS framework consisting of –Si–O– is highly heat-stable. In addition, when crosslinking octavinyl-POSS in a linear polymer, POSS can transform the linear polymer into a three-dimensional network. Therefore, the polymer’s thermal and mechanical stability can be improved. However, excessive amounts of vinyl groups inside the LSR cause over-curing, thus resulting in brittleness as well as poor elasticity and mechanical properties. Therefore, the Si–H/Si–vinyl ratio in a silicone curing system is important for determining the various properties of silicone [29,30]. The Si–H/Si–vinyl ratio must be considered when T-8 POSS with vinyl groups is used as a crosslinking agent.

Herein, four types of POSS-V with different amounts of POSS vinyl groups were synthesized for application as crosslinking agents in F-LSRs. The percentages of vinyl groups in each POSS were 100, 75, 50, and 0%, named POSS-V8, POSS-V6, POSS-V4, and POSS-V0, respectively. The main chain and crosslinker were directly synthesized and named F-silicone and F-crosslinker, respectively. Subsequently, F-LSR was prepared by connecting F-silicone as the main chain with F-crosslinker and POSS as crosslinking agents. The mechanical and thermal properties of the F-LSR prepared by changing the type and content of POSS-V with various vinyl groups were characterized using analytical tools, including Fourier transform infrared spectroscopy (FT-IR), NMR, scanning electron microscopy (SEM), matrix-assisted laser desorption/ionization time-of-flight mass spectroscopy (MALDI-ToF MS), X-ray diffraction (XRD), UTM, differential scanning calorimetry (DSC), dynamic mechanical analysis (DMA), and thermogravimetric analysis (TGA).

## 2. Materials and Methods

### 2.1. Materials

All chemicals and solvents were commercially available and used without further purification. Tetramethylammonium hydroxide (TMAH, 25% in water) and methanol were purchased from Alfa Aesar (Haverhill, MA, USA). Octamethylcyclotetrasiloxane (D_4_, >99%), tetramethyltetravinylcyclotetrasiloxane (Vi-D_4_, >99%), divinyltetramethyldisiloxane (ViVi, >99%), and hexamethyldisiloxane (MM, >99%) were purchased from DAMI POLYCHEM (Iksan, Republic of Korea). Meanwhile, 1,3,5-trimethyl-1,3,5-tris(3,3,3-trifluoropropyl)cyclotrisiloxane (F-D_3_, >99%) was purchased from the HRS Corporation (Seoul, Republic of Korea). To prepare the liquid silicone, fumed silica was purchased from Grace Continental Korea (Bucheon, Republic of Korea).

### 2.2. F-Silicone and F-Crosslinker Synthesis

Raw F-silicone was synthesized in a 1 L four-neck flask with a mechanical stirrer and reflux condenser. Before initiating polymerization, 0.882 mol D_4_ (261.07 g), 0.882 mol F-D_3_ (413.25 g), and 0.036 mol Vi-D_4_ (12.40 g) were charged into a flask under an argon atmosphere at 90 °C for 1 h to maintain the initiating temperature. Next, 0.3 wt% tetramethylammonium silanolate (TMAS, 2.06 g) and 0.006 mol ViVi (1.116 g) were added to the solution and stirred at 300 rpm under an argon atmosphere for 5 h. After the reaction, the product was heated to 150 °C and maintained for 24 h under vacuum to remove unreacted reactants and initiators. Finally, the products were purified with methanol to eliminate cyclic monomers. The purified F-silicone was a transparent, high-viscosity liquid obtained with a yield of 85%.

The F-crosslinker was synthesized in a 1 L four-neck flask with a mechanical stirrer and reflux condenser. First, D_4_ and F-D_3_ were charged into a flask under an argon atmosphere at 90 °C for 1 h to maintain the initiating temperature. The reaction was initiated by adding TMAS to the reactor under stirring at 300 rpm and 90 °C. After initiating the reaction, H-D_4_ was added dropwise to this solution over 0.5 h under vigorous stirring using a 250 mL dropping funnel. Subsequently, the end-blocker MM was added to the reactor, and the mixture was stirred at 90 °C for 2 h. After the reaction, the products were heated to 150 °C and maintained under vacuum for 24 h to remove unreacted reactants and initiators. Finally, the products were purified with methanol to eliminate the cyclic monomers.

### 2.3. Synthesis of POSS-Vs

An acetone (500 g) solution containing a mixture (0.45 mol) of VTMS and MTMS was charged into a 1 L four-neck flask equipped with a mechanical stirrer. The solution was stirred at 300 rpm under an argon atmosphere at 40 °C. HCl was added dropwise to this solution over 2 h under vigorous stirring using a 250 mL dropping funnel. Next, the mixture was stirred at 40 °C for 34 h and subsequently cooled to room temperature. After the reaction, a white solid was deposited on the flask wall. The white powder was filtered and washed with copious amounts of ethanol, water, and acetone and subsequently dried in an air circulation oven at 60 °C overnight. The crude product was recrystallized from a mixture (300 mL) of dichloromethane and acetone (*v*/*v* 1:3) and allowed to stand for 24 h at room temperature. The recrystallized POSS was collected by filtration using filter paper and dried overnight in an air circulation oven at 60 °C. The purified POSSs were obtained as white powders in 30–40% yields. All synthesized POSS-Vs had vinyl content of 100, 75, 50, and 0%, named POSS-V8, POSS-V6, POSS-V4, and POSS-V0, respectively.

### 2.4. Preparing F-LSR-POSS

All prepared POSS-Vs were homogenously dissolved in 30 mL of THF in a 500 mL Teflon beaker (DAIHAN SCIENTIFIC CO, Wonju, Republic of Korea) to ensure complete dispersion. Next, raw F-silicone and F-crosslinker were added successively to the beaker and mixed vigorously using a hand mixer. The amount of reactant was adjusted such that the ratio of Si–H/Si–Vi in the product was maintained at 1, and the total amount was 300 g. The mixture was then placed in a vacuum oven (DAIHAN SCIENTIFIC CO, Wonju, Republic of Korea) at 100 °C for 2 h to remove residual moisture and air bubbles. Subsequently, 0.1 g of Karstedt’s catalyst was mixed using a hand mixer (DAIHAN SCIENTIFIC CO, Wonju, Republic of Korea) at 300 rpm and directly injected into the molding cavity for hot-press molding. All the F-LSR-POSSs were compression-molded at 140 °C for 5 min and post-cured at 200 °C for 4 h in an oven. The formulations of all synthesized F-LSR-POSSs are presented in Table 1.

### 2.5. Characterization

Fourier transform infrared spectroscopy (FT-IR) was used to analyze the functional groups of the prepared POSS-Vs. FT-IR spectra were obtained using a VERTEX 80 V spectrometer (Bruker, Karlsruhe, Germany) with a resolution of 1 cm^−1^ by acquiring 32 scans under vacuum (pellet composed of KBr and substrate at a ratio of 100:1). The analysis was conducted within the frequency range of 4000–400 cm^−1^.

^1^H-NMR at 400 MHz (Bruker Advance III spectrometer (Bruker, Karlsruhe, Germany)) was performed to elucidate the detailed chemical structure and calculate the ratio of each functional group of the sample. All samples were prepared in 0.6 mL of CDCl3, and the NMR spectra were recorded.

Attenuated total reflection Fourier transform infrared spectroscopy (ATR-FTIR) spectra were obtained using a Spectrum 2 spectrometer (PerkinElmer, Waltham, MA, USA) with a resolution of 1 cm^−1^ by acquiring 16 scans. The analysis was performed within a frequency range of 4000–400 cm^−1^.

SEM (S-4300, Hitachi, Tokyo, Japan) was used to observe the morphology of the POSS-Vs. Elemental mapping was performed using energy-dispersive spectroscopy (EDS; S-4300, Hitachi, Tokyo, Japan).

MALDI-ToF MS was performed using an Autoflex maX (Bruker, Karlsruhe, Germany) instrument equipped with a 355 nm ND:YAG laser in the positive ion reflection mode, with 1,8,9-anthracenetriol as the matrix and silver trifluoroacetate (AgTFA) as the ion source. The samples were prepared by mixing dithranol (10 mg/mL in THF), the sample (1 mg/mL in THF), and AgTFA. Electrospray ionization of the samples was performed at a capillary voltage of 3 kV.

The crystal structures of the F-LSR-POSSs were determined by XRD (X’Pert PRO, Philips, Amsterdam, Netherland) using a Cu Kα radiation generator within the 2θ range of 5°–90°.

Tensile tests of the F-LSR-POSSs were performed according to the ASTM D-412 method using a universal testing machine. All test specimens were dumbbell-type 1 at room temperature with a crosshead speed of 500 mm/min. The hardness of the F-LSR was analyzed according to ASTM D2240-15el using an ASKER Durometer type A (KOBUNSHI KEIKI CO, Kyoto, Japan).

The storage and loss moduli of the F-LSR-POSSs were measured via DMA using a dynamic mechanical analyzer in tensile mode under an argon atmosphere. All samples were scanned from −130 to 30 °C at a heating rate using a frequency of 1 Hz.

DSC (DSC 8000 Perkin Elmer, Waltham, MA, USA) was used to investigate the thermal properties at low temperatures. DSC measurements of all samples were performed using 5–10 mg of the sample from −130 to 30 °C, at heating and cooling rates of 10 °C/min, under an argon atmosphere.

TGA was carried out using a TGA 4000 instrument (PerkinElmer, Waltham, MA, USA), wherein the samples were heated from 40 to 750 °C at 10 °C/min under a nitrogen atmosphere. The weight loss rate curves of all samples were obtained by differentiating the TGA thermograms.

## 3. Results and Discussion

**Scheme 1:**
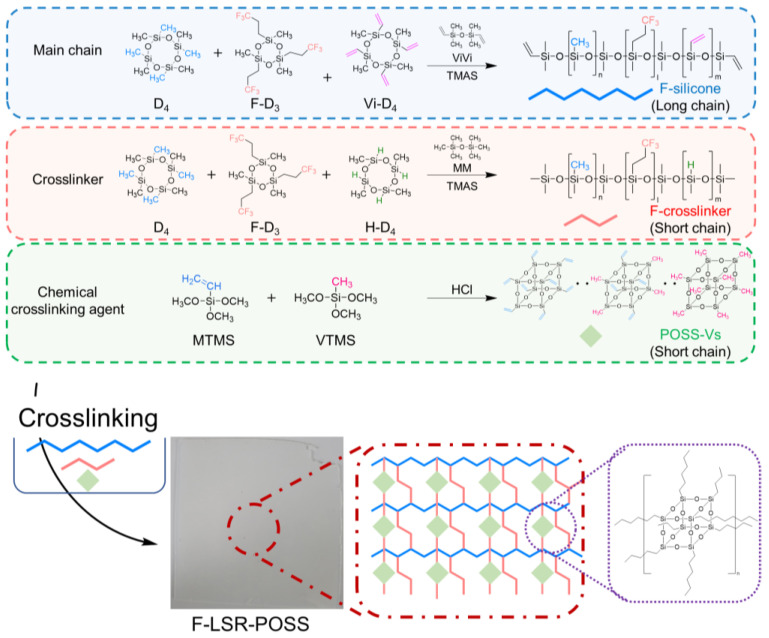
Schematic of the F-LSR-POSSs.

The entire synthetic process of F-LSR-POSS is illustrated in Scheme 1.

### 3.1. Synthesis of F-Silicone and F-Crosslinker

Prior to preparing the F-LSR-POSSs, F-silicone and F-crosslinkers were synthesized. F-silicone is a long-chain silicone composed of methyl, trifluoropropyl, and vinyl groups, whereas the F-crosslinker is a short-chain silicone composed of methyl, trifluoropropyl, and hydrido groups. Detailed information regarding the F-silicone and F-crosslinker is provided in Table S1. Figure S1a shows the FT-IR spectra of F-silicone and F-crosslinker. The strong absorption band from 1130 to 1000 cm^−1^ in all spectra was attributed to the–Si–O–Si– asymmetric stretching vibration of the F-silicone and F-crosslinker. In addition, the absorption bands of –CF_3_ (1210 cm^−1^), Si–CH_3_ (1226 cm^−1^), the –CH bond in –CH_3_ (2960 cm^−1^), the −CH_2_−CH_2_− bond (1315 cm^−1^), and the C−H bond in −CH_2_− (1128 cm^−1^) were observed in the spectra of the F-silicone and F-crosslinker. A characteristic absorption band at 2245 cm^−1^ for the Si–H bond was observed in the FT-IR spectrum of the F-crosslinker [31,32,33]. However, the absorption band arising from the CH_2_=CH_2_ bond in the F-silicone FT-IR spectrum was difficult to identify because of the small amount of CH_2_=CH_2_ present. In the NMR spectra of the F-silicone and F-crosslinker, a peak corresponding to Si–CH_3_ at approximately 0 ppm was confirmed (Figure S1). In addition, peaks at 0.7 and 2.1 ppm characteristic of Si–CH_2_CH_2_CF_3_ were clearly identified in both NMR spectra. Furthermore, several peaks at approximately 6 ppm corresponding to Si-vinyl were confirmed for the F-silicone, and a peak at 4.7 ppm corresponding to Si–H was confirmed for the F-crosslinker [34]. From the FT-IR and NMR spectra, all functional groups in the F-silicone and F-crosslinker were confirmed, and the amounts of Si-vinyl groups in F-silicone and Si–H groups in the F-crosslinker were determined to be 1.25 and 10.97 mol%, respectively.

### 3.2. Synthesis and Characterization of POSS-Vs

Four types of T8-POSS with various vinyl ratios were synthesized via cohydrolysis–cocondensation. The four POSS-Vs with vinyl group content of 100, 75, 50, and 0% were named POSS-V8, POSS-V6, POSS-V4, and POSS-V0, respectively (Table S2). Figure S2 shows a single strong absorption band attributable to –Si–O–Si– in the cage structure at 1110 cm^−1^ in all the POSS spectra. The peak intensities of the –C=C– group at 1000 and 1616 cm^−1^ gradually decreased from POSS-V8 to POSS-V0 because of the decreased number of vinyl groups (Figure S2a) [35,36]. By contrast, the Si–CH_3_ peak at 824 cm^−1^ became distinguishable as the number of methyl groups increased. Figure S2b shows the ^1^H-NMR spectra of all the POSS-Vs. As the number of vinyl groups inside the POSS increased, the –CH=CH_2_ peak at 6 ppm gradually increased and that of the –CH_3_ peak at 0 ppm decreased. SEM microphotographs of the POSS samples are displayed in Figure S3; evidently, all POSSs possessed a cuboid structure, similar to that reported previously [37,38]. Finally, the molecular weight of T8-POSS was measured (Figure S4). As a result, the structure of all POSS-V was T-8 POSS, and although it was a mixture of different vinyl groups, the ratio of total vinyl groups was 100, 75, 50, and 0%, respectively.

### 3.3. Characterization of F-LSR-POSS

**Figure 1:**
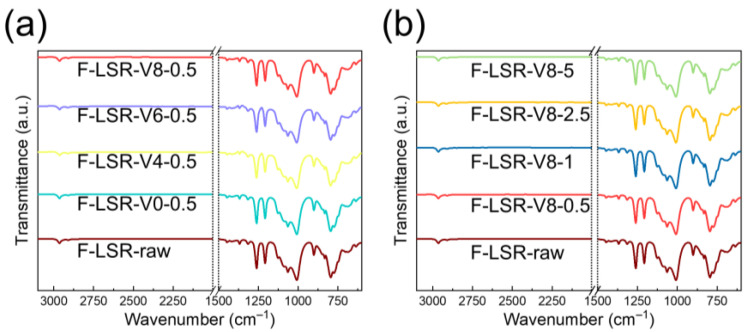
FT-IR spectra of the prepared F-LSR-POSSs with (**a**) various types of POSS-Vs and (**b**) different amounts of POSS-V8.

In F-LSR-POSS, the ratio of Si–H/Si–CH=CH_2_ was set to 1 to ensure that all F-LSR-POSSs had the same crosslinking site. To meet this goal, the ratio of the F-crosslinker to F-silicone in the F-LSR was changed according to the type of POSS loaded. In Figure 1, the FT-IR spectra of the F-LSR-POSSs are provided for functional group characterization. Most spectral shapes of the F-LSR-POSSs were similar to those of F-silicone, and all F-LSR-POSSs were fully crosslinked, as confirmed by the disappearance of the Si–H group absorption band at 2000 cm^−1^.

**Figure 2:**
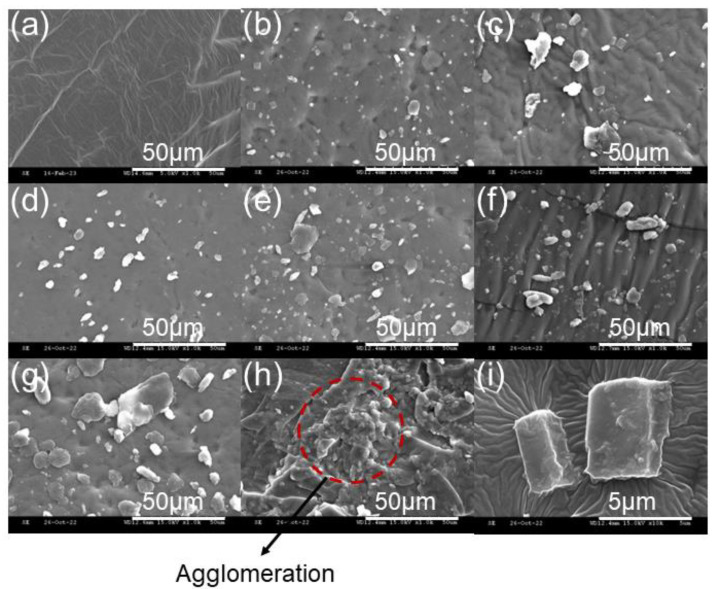
SEM microphotographs of the F-LSR-POSSs: (**a**) F-LSR-raw, (**b**) F-LSR-V0-0.5, (**c**) F-LSR-V4-0.5, (**d**) F-LSR-V6-0.5, (**e**) F-LSR-V8-0.5, (**f**) F-LSR-V8-1, (**g**) F-LSR-V8-2.5, (**h**) F-LSR-V8-5, and (**i**) magnified crosslinked POSS.

Figure 2 shows SEM microphotographs of the F-LSR-POSS sample with 0.5 wt% POSS-V with different vinyl ratios. At the 0.5 wt% POSS loading, POSS was well distributed in the F-LSR matrices regardless of the vinyl group number. However, with increasing amounts of POSS-V8 from 0.5 to 5 phr, agglomerated POSSs gradually appeared (Figure 2). An agglomeration of POSS-V8, shown in Figure 2h, indicated that POSS-V8 was poorly dispersed in the F-LSR matrix. This phenomenon was ascribed to the large trifluoropropyl groups in F-LSR-POSSs, which hindered the uniform dispersion of POSS-V owing to their high polarity and steric bulkiness. Therefore, with increasing POSS content in the F-LSR-POSSs, phase separation between POSS-V and the F-LSR matrix occurred, facilitated by the presence of numerous trifluoropropyl groups in F-LSR-POSSs with some POSS-V agglomeration.

**Figure 3:**
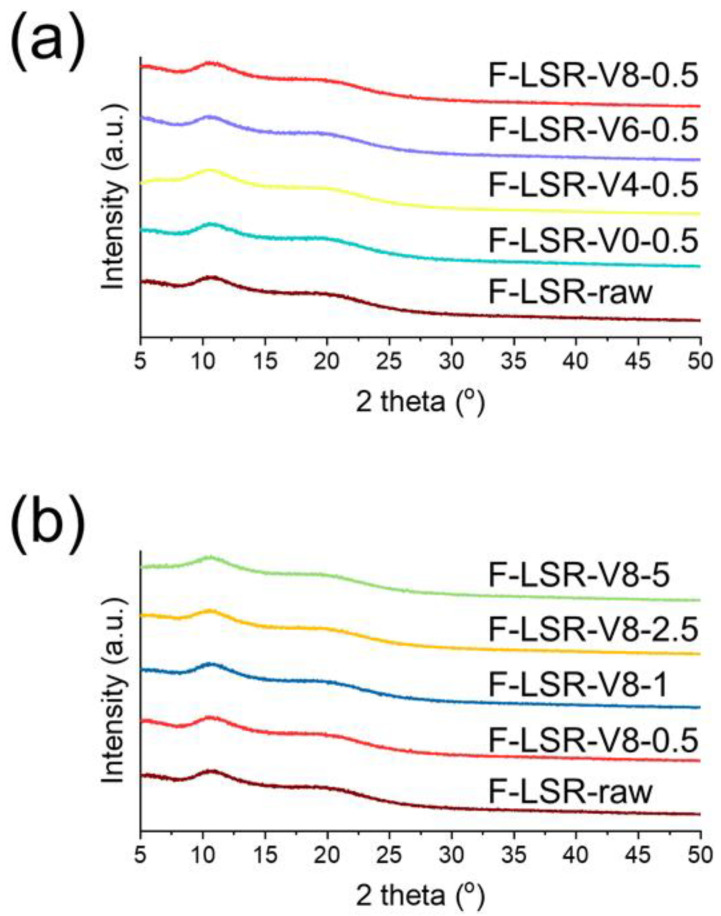
XRD patterns of the POSS-Vs with (**a**) various types of POSSs and (**b**) different amounts of POSS-V8.

XRD analysis was performed to determine the synthesized F-LSR-POSSs’ crystallinity. The XRD diffractograms of the F-LSR-POSSs revealed similar broad curves, thus indicating an amorphous nature (Figure 3). Generally, POSS-V has high crystallinity [38,39]; however, the specific crystallization peak was absent in most diffractograms of F-LSR-POSS because almost POSSs were well dispersed in the F-silicone matrix. Although SEM displayed that POSS-V agglomeration occurred when 5 wt% of POSS-V was introduced to F-LSR, it was not sufficient to affect the crystallinity of F-LSR.

### 3.4. Mechanical Properties of the F-LSR-POSSs

**Figure 4:**
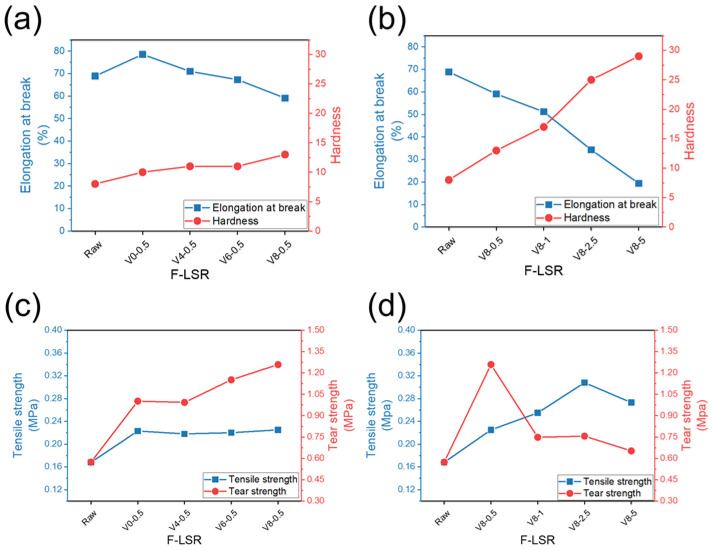
Mechanical properties of the F-LSR-POSSs: (**a**,**b**) elongation and hardness of the F-LSR-POSSs and (**c**,**d**) tensile and tear strengths of the F-LSR-POSSs.

Mechanical properties including tensile strength, tear strength, elongation at break, and hardness were identified using a UTM and durometer. Figure 4 and Table 2 present the mechanical properties of the F-LSR-POSSs, and the hardness and elongation of the F-LSR-POSSs are shown in Figure 4a,b. As the vinyl group ratio in POSS-V increased, the elongation of the F-LSR-POSSs decreased and their hardness increased. This is because the additional vinyl groups in POSS-V caused more chemical crosslinking by the hydrosilylation of F-LSR-POSS, thus resulting in higher crosslinking densities. By contrast, the hardness and elongation of F-LSR-V0-0.5 increased compared with the F-LSR-raw because POSS-V0 without vinyl groups acts as a physical filler, similar to the silica in F-LSR-V0-0.5. As shown in Figure 4b, with increasing amounts of POSS-V8 in F-LSR-POSS, the hardness increased and elongation decreased, consistent with previous results. In particular, the changes in the elongation and hardness of the F-LSR-POSS were substantial because the higher amount of POSS-V8 in F-LSR-POSSs increased the crosslinking density and the strong inorganic backbone of POSS in the F-LSR.

Meanwhile, the tensile and tear strengths increased compared with those of F-LSR-raw when POSS was employed as a crosslinking agent. The effects of the type or loading of POSS on the tensile and tear strengths could not be identified because numerous factors affect the mechanical properties (Figure 4c,d) [40,41,42,43]. The F-LSR-POSSs with the highest tensile and tear strengths were F-LSR-V8-0.5 and F-LSR-V8-2.5, respectively.

### 3.5. Viscoelastic Properties and Crosslinking Density of the F-LSR-POSSs

**Figure 5:**
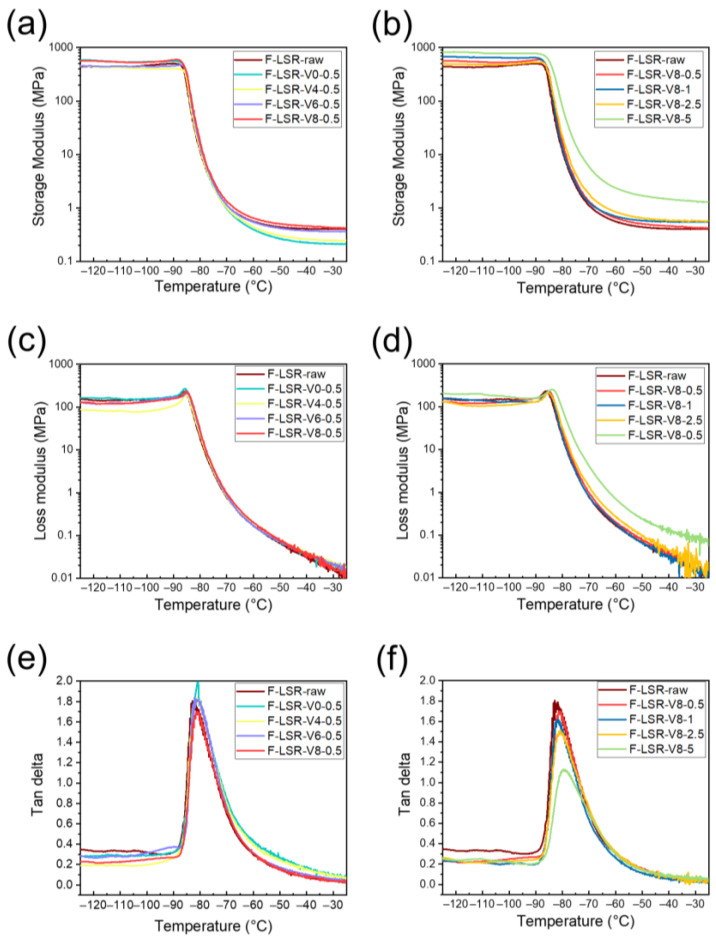
DMA analysis of all F-LSR-POSSs: (**a**,**b**) storage modulus; (**c**,**d**) loss modulus; and (**e**,**f**) tan delta.

DMA analysis of the F-LSR-POSSs was performed to investigate the effect on the viscoelastic performance upon introducing POSS-V as a chemical crosslinking agent at low temperatures. The storage modulus (*E*′), loss modulus (*E*′), and tan δ of the F-LSR-POSSs are shown in Figure 5. In the DMA analysis, each tan δ peak indicating *T_g_* was measured at approximately −81 °C, thus exhibiting similar viscoelastic behavior as fluorosilicone, as reported previously [44]. As shown in Figure 5, the E′ and E″ values of all F-LSR-POSSs decreased rapidly after *T_g_*. Interestingly, the tan δ value at *T_g_* decreased with increasing vinyl content of POSS-V and increasing POSS-V8 loading in the F-LSR-POSSs (Figure 5e,f). As the vinyl group content originating from POSS-Vs in F-LSR-POSSs increased, the elastic behavior gradually increased instead of the viscous behavior. The number of crosslinking sites increased owing to the high POSS loading, thereby reducing the friction between molecular chains and accumulating more elastic energy [45].

**Figure 6:**
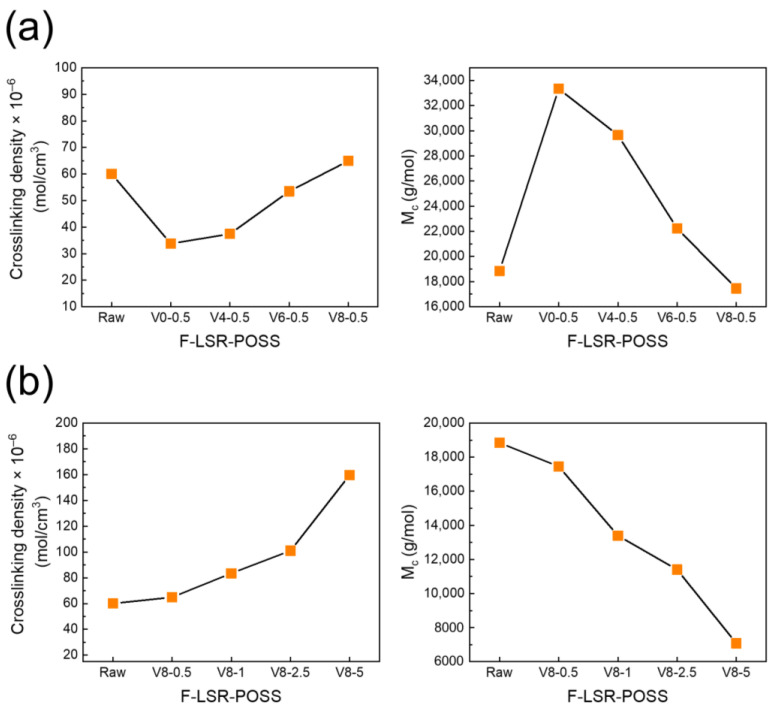
Crosslinking densities and *M_c_* of the F-LSR-POSSs: (**a**) with various types of POSSs and (**b**) with different POSS-V8 loadings.

However, the storage modulus value at the rubbery plateau (*T* > *T_g_*) is proportional to the crosslinking density [43]. All F-LSR-POSS crosslinking densities were calculated by the storage modulus measured by DMA in the rubbery plateau region using the modulus of elasticity method. The following formula describing the modulus of elasticity was proposed by Lakshimi et al. [46,47,48].

(1) $$\sigma =\frac{E^{\prime }}{3RT}$$

where *σ* is the crosslinking density of the F-LSR-POSSs; *R* is the gas constant; *T* is the absolute temperature; and *E′* is the elastic modulus of the F-LSR-POSSs in the rubbery plateau region. In addition, the molecular weight between the crosslinking sites can be defined using the following formula:

(2) $$M_{c}=\frac{3\rho RT}{E^{\prime }}$$

where *M_c_* is the molecular weight between the crosslinking sites of the F-LSR-POSSs; and *ρ* is the F-LSR-POSS density. The crosslinking densities and molecular weights of the crosslinking sites are presented in Table 3 and Figure 6. Evidently from Table 3, all densities ranged between 1.11 and 1.19 g/cm^3^, which is identical to that previously reported for fluorosilicone rubber [43]. Figure 6a confirms that the crosslinking density of F-LSR-V0-0.5 decreased compared with that of the F-LSR-raw. By contrast, the *M_c_* of F-LSR-V0-0.5 increased from 18,841 to 33,332 g/mol compared with that of the F-LSR-raw. This indicates that POSS-V0 cannot be chemically crosslinked with silicone because it contains only methyl groups, without vinyl groups. Therefore, POSS-V0 acts as a physical filler, thereby reducing the crosslinking density and increasing the *M_c_* of the F-LSR. Conversely, at constant POSS-V loading, the increasing vinyl group content of the POSS-V increased the crosslinking density of F-LSR-POSS while decreasing *M_c_*. With the increasing vinyl content of POSS-V, the number of hydrosilylation chemical crosslinking sites with F-crosslinker increased, and the crosslinking density of F-LSR-POSS increased. Furthermore, with increasing numbers of chemical crosslinking points, the dense crosslinking points resulted in a decreased *M_c_*. Figure 6b shows the crosslinking density and *M_c_* of F-LSR-POSS with different POSS-V8 loadings. Evidently, with increasing POSS-V8 content, the crosslinking density increased significantly as *M_c_* decreased. This is consistent with the previous results that reported that the crosslinking density increases owing to an increase in vinyl group content. Therefore, with increasing POSS-V loading, the crosslinking density of the resulting F-LSR-POSSs also increases. Further, POSS-V with vinyl groups as a chemical crosslinking agent was confirmed to significantly affect the crosslinking density of the F-LSR-POSSs.

### 3.6. Thermal Properties of the F-LSR-POSSs

**Figure 7:**
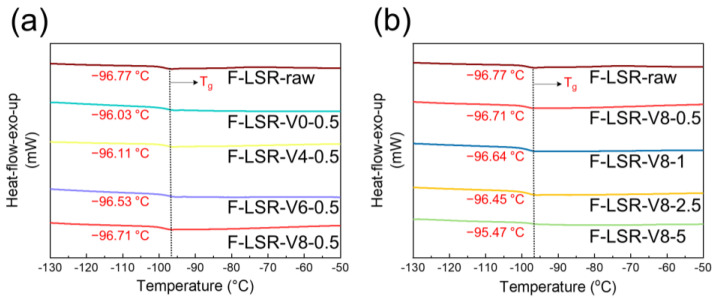
DSC curves of the F-LSR-POSSs with (**a**) various types of POSSs and (**b**) different POSS-V8 loadings.

The thermal properties of the F-LSR-POSSs at high and low temperatures were analyzed using DSC, DMA, and TGA (Table 4). The *T_g_* of the F-LSR-raw was measured at −96.6 °C via DSC analysis (Figure 7) and −81.2 °C via DMA analysis. A discrepancy of approximately 15 °C was observed in the *T_g_* measured by DSC compared with DMA owing to the difference in the measurement method [49]. Generally, crystalline regions do not form in F-LSR because the trifluoropropyl group breaks the symmetry and regularity of the polysiloxane monomer [31,50]. Therefore, all F-LSR-POSSs also exhibit a glass transition temperature without a melting temperature. In DSC analysis, the *T_g_* of all F-LSR-POSSs with different POSS-V loadings was around −96 °C. Similarly, the *T_g_* of all F-LSR-POSSs measured by DMA was approximately −81 °C. Therefore, the *T_g_* values of the F-LSR-POSSs did not change significantly with varying vinyl group ratios and POSS-V loading. Thus, the introduction of POSS-V as a chemical crosslinking agent into F-LSR-POSS did not significantly change the thermal properties at low temperatures and had little effect on the cold resistance of the F-LSR-POSSs.

**Figure 8:**
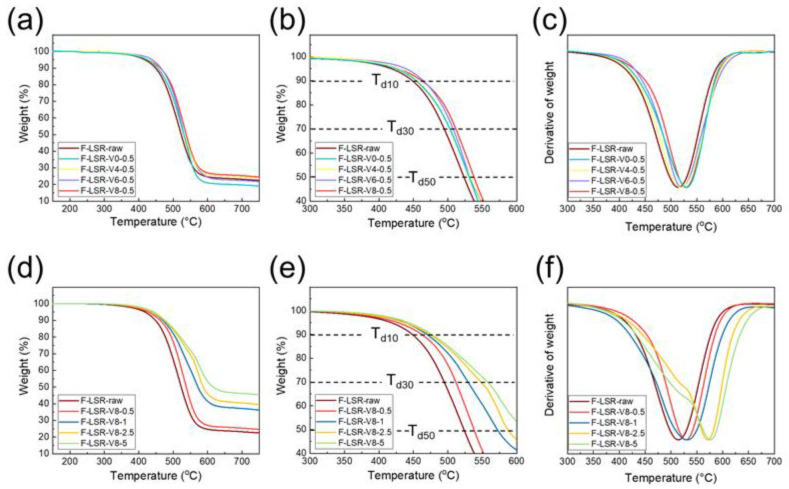
TGA analysis of the F-LSR-POSSs: (**a**,**b**) TGA thermograms of the F-LSR-POSSs with various types of POSSs; (**c**) DTGA of F-LSR-POSSs with various types of POSSs; (**d**,**e**) TGA thermograms of F-LSR-POSSs with different loadings of POSS-V8; and (**f**) DTGA of F-LSR-POSSs with different loadings of POSS-V8.

TGA was performed to determine the effect of POSS-V on the thermal properties at high temperatures, and Figure 8 shows the TGA thermograms of the F-LSR-POSSs. The deterioration point was classified into four types to accurately analyze the heat resistance. The temperatures at 10, 30, and 50% weight loss are denoted as T_d10_, T_d30_, and T_d50_, respectively, and T_dmax_ denotes the fastest mass loss rate in the DTG curve (Figure 8). As shown in Figure 8a, the T_d10_, T_d30_, and T_d50_ of the F-LSR-raw were determined to be 448.5, 494.7, and 523.2 °C, respectively, whereas T_dmax_ was observed at 514.19 °C. The degradation points of all F-LSR-POSSs were confirmed to shift to higher temperatures compared with the F-LSR-raw. Two factors increased the heat resistance of F-LSR-POSSs when POSS-V was introduced. First, the high-density chemical crosslinking owing to the increased vinyl group content strengthened the bonding between silicone chains. Second, the framework structure of POSS-V, which has a higher ratio of inorganic Si–O bonds than silicone, can increase the heat resistance of the F-LSR-POSSs. Increasing the vinyl group content of POSS-V is speculated to increase the number of chemical crosslinking sites, thus resulting in improved heat resistance. Meanwhile, with increased POSS loading, the degradation of the F-LSR-POSSs shifted to considerably higher temperatures (Figure 8b). In particular, the T_d10_, T_d30_, T_d50_, and T_dmax_ values of F-LSR-V8-0.5 were 454.00, 500.94, 530.76, and 530.27 °C, respectively; these are significantly higher than those of the F-LSR-raw. Remarkably, T_d50_ increased by >90 °C when only 5 wt% POSS-V8 was added, thus indicating that the heat resistance was considerably improved with POSS loading. Interestingly, with further increasing POSS-V8 content, the amount of residue >700 °C increased. Generally, silicone rubber can be converted to silica at high temperatures and chemically crosslinked POSS can accelerate this phenomenon [51]. Consequently, the heat resistance improved when POSS was crosslinked to the F-LSR matrix, which enhanced the thermal stability at high temperatures.

## 4. Conclusions

F-LSR-POSSs were prepared by introducing POSS-V with vinyl groups as a chemical crosslinking agent to improve the heat resistance of fluorosilicone, which has excellent cold resistance but poor heat resistance compared with general PDMS. First, F-silicone, F-crosslinker, and various POSSs required for the fabrication of F-LSR-POSS were successfully synthesized and characterized. Most POSSs were well dispersed within the F-LSR via chemical crosslinking. When the loading of POSS-V8 exceeded 5 wt%, phase separation occurred between F-LSR and POSS owing to the large amount of trifluoropropyl groups in F-LSR, as confirmed by XRD and SEM. In terms of mechanical properties, the elongation and hardness consistently exhibited opposite trends with varying POSS loadings or types of POSS. Although the mechanical properties improved upon POSS introduction, no defined correlation between the tear and tensile strengths and the type or loading of POSS-V could be established. DMA analysis confirmed the storage modulus, loss modulus, and tan δ of POSS-V with different vinyl content. In addition, higher ratios of vinyl groups in POSS-V and higher POSS-V8 loadings increased the crosslinking density of the resulting F-LSR-POSSs. *T_g_* values were identified through DSC and DMA analyses, and the cold resistances of the prepared F-LSR-POSSs were verified. Although all *T_g_* values of the F-LSR-POSSs were slightly changed upon POSS introduction, the fluctuation range was within 2 °C and it did not significantly affect the cold resistance. However, the heat resistance of the F-LSR was dramatically changed upon POSS introduction and the T_d50_ was improved by >90 °C when only 5 wt% of POSS-V8 was added. This indicated that POSS with various vinyl groups could be introduced into F-LSR as a crosslinking agent and successfully crosslinked in the LSR matrix. The low mechanical strength and heat resistance of fluorosilicone could be improved by POSS introduction. This study clearly established strategies to increase the operating temperature of fluorosilicone and expand its application potential to harsh conditions.

## Figures and Tables

**Table 1:** Reactant compositions for the preparation of FVMQs.

Sample	F-LSR (g)	POSS-V		Mol Ratio of Si–H/Si–Vinyl
		Type	Amount (phr)	
F-LSR-raw	300	/	/	1
F-LSR-V0-0.5		POSS-V0	0.5	
F-LSR-V4-0.5		POSS-V4	0.5	
F-LSR-V6-0.5		POSS-V6	0.5	
F-LSR-V8-0.5		POSS-V8	0.5	
F-LSR-V8-1		POSS-V8	1	
F-LSR-V8-2.5		POSS-V8	2.5	
F-LSR-V8-5		POSS-V8	5	

**Table 2:** Mechanical properties of the F-LSR-POSSs.

F-LSR-POSSs	Tensile Strength (MPa)	Tear Strength (MPa)	Elongation at Break (%)	Hardness
F-LSR-raw	0.168	0.573	68.9	8
F-LSR-V0-0.5	0.223	1.002	78.5	10
F-LSR-V4-0.5	0.218	0.993	71	11
F-LSR-V6-0.5	0.178	1.152	67.3	11
F-LSR-V8-0.5	0.225	1.259	59.1	13
F-LSR-raw	0.168	0.573	68.9	8
F-LSR-V8-0.5	0.225	1.259	59.1	13
F-LSR-V8-1	0.255	0.748	51.2	17
F-LSR-V8-2.5	0.308	0.556	34.3	25
F-LSR-V8-5	0.273	0.552	19.4	29

**Table 3:** Densities, crosslinking densities, and *M_c_* values for the F-LSR-POSSs.

Sample	Density (g/cm^3^)	Crosslinking Density ×10^−6^ (mol/cm^3^)	*M_c_* (g/mol)
F-LSR-raw	1.132	60.079	18,841.870
F-LSR-V0-0.5	1.125	33.751	33,332.627
F-LSR-V4-0.5	1.110	37.435	29,651.351
F-LSR-V6-0.5	1.188	53.463	22,220.857
F-LSR-V8-0.5	1.133	64.920	17,452.329
F-LSR-raw	1.132	60.079	18,841.870
F-LSR-V8-0.5	1.133	64.920	17,452.329
F-LSR-V8-1	1.118	83.476	13,393.092
F-LSR-V8-2.5	1.151	100.983	11,397.934
F-LSR-V8-5	1.130	159.744	7073.800

**Table 4:** Thermal properties of the F-LSR-POSSs obtained from DSC, DMA, and TGA analyses.

Sample	*T_g_* (DSC) (°C)	*T_g_* (DMA) (°C)	T_d10_ (°C)	T_d30_ (°C)	T_d50_ (°C)	T_dmax_ (°C)	Residue at 700 °C (wt%)
F-LSR-raw	−96.8	−81.7	448.5	494.7	523.2	514.2	23.2
F-LSR-V0-0.5	−96.0	−79.4	454.0	500.9	530.8	530.3	19.8
F-LSR-V4-0.5	−96.1	−80.9	455.4	502.5	531.6	519.1	24.5
F-LSR-V6-0.5	−96.6	−81.5	460.3	507.5	532.2	520.8	22.4
F-LSR-V8-0.5	−96.7	−81.7	461.9	511.5	537.9	529.0	25.3
F-LSR-raw	−96.8	−81.7	448.5	494.7	523.2	514.2	23.2
F-LSR-V8-0.5	−96.7	−81.7	461.9	511.5	537.9	529.0	25.3
F-LSR-V8-1	−96.6	−80.5	469.4	529.2	571.7	531.3	37.2
F-LSR-V8-2.5	−96.5-	−81.1	473.0	548.0	587.0	571.2	40.5
F-LSR-V8-5	−95.5	−80.7	476.4	557.3	616.6	576.2	46.2

## Data Availability

Not applicable.

## Supplementary Materials

The following supporting information can be downloaded at https://www.mdpi.com/article/10.3390/polym15051300/s1, Table S1: Functional groups and viscosity data of F-silicone and F-crosslinker; Figure S1: (a) FT-IR spectra of F-silicone and F-crosslinker and (b) ^1^H-NMR spectra of F-silicone and F-crosslinker; Table S2: Formulation of the synthesized POSS-Vs; Figure S2: (a) FT-IR spectra of synthesized POSS-Vs; (b) ^1^H-NMR spectra of synthesized POSS-Vs; Figure S3: SEM micrographs of the POSS-Vs: (a,b) POSS-V8; (c,d) POSS-V6; (e,f) POSS-V4; (g,h) POSS-V0; Figure S4: MALDI-TOF MS spectra of all POSS-Vs.
