# Supplementary material for: Improvement of Heat Resistance of Fluorosilicone Rubber Employing Vinyl-Functionalized POSS as a Chemical Crosslinking Agent
Source: Polymers (Basel). 2023 Mar 4;15(5):1300. doi: 10.3390/polym15051300 (PMC10007170; doi:10.3390/polym15051300)
Supplement: Supplementary file 1 [file polymers-15-01300-s001.zip › polymers-2166249-supplementary.pdf]

polymers

MDPI

Article

Supporting Information

Improvement of heat resistance of fluorosilicone rubber employing vinyl-functionalized POSS as a chemical crosslinking agent

Jae Il So, Chung Soo Lee, Byeong Seok Kim, Hyeon Woo Jeong, Jin Sung Seo, Sung-Hyeon Baeck, Sang Eun Shim*, and Yingjie Qian*

Table S1 Functional groups and viscosity data of the F-silicone and F-crosslinker.

Raw material	Structure	Functional group ratio (mol %)				Viscosity (cP)
		DM-block	MF-block	MV-block	MH-block	
F-silicone	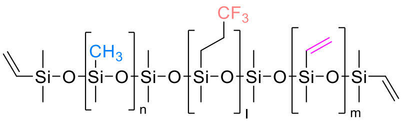	55.64	43.11	1.25		12200
F-crosslinker	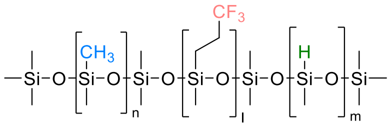	54.94	34.09		10.97	970

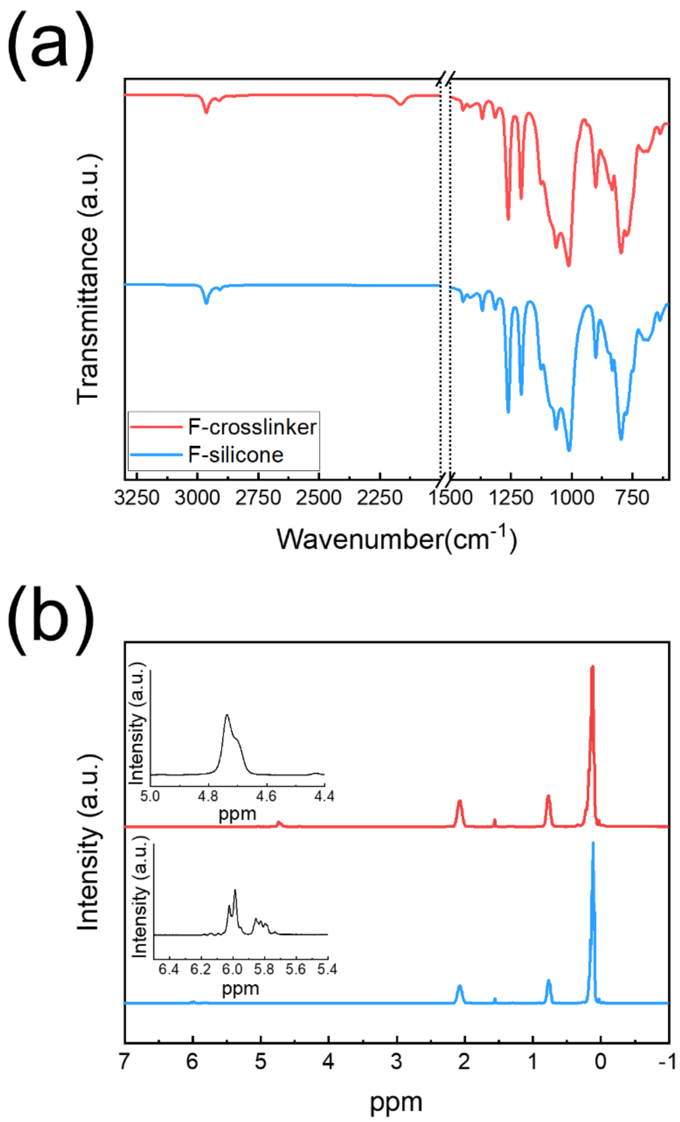

Figure S1. (a) FT-IR spectra of the F-silicone and F-crosslinker and (b) ^{1}H-NMR spectra of the F-silicone and F-crosslinker.

Table S2. Formulation of the synthesized POSS-Vs.

Material	VTMS [molar ratio]	MTMS [molar ratio]	5 M HCl solution [molar ratio]	Theoretical vinyl content [mol%]
POSS-V0	0	1	6	0
POSS-V4	0.5	0.5	27	50
POSS-V6	0.75	0.25	27	75
POSS-V8	1	0	27	100

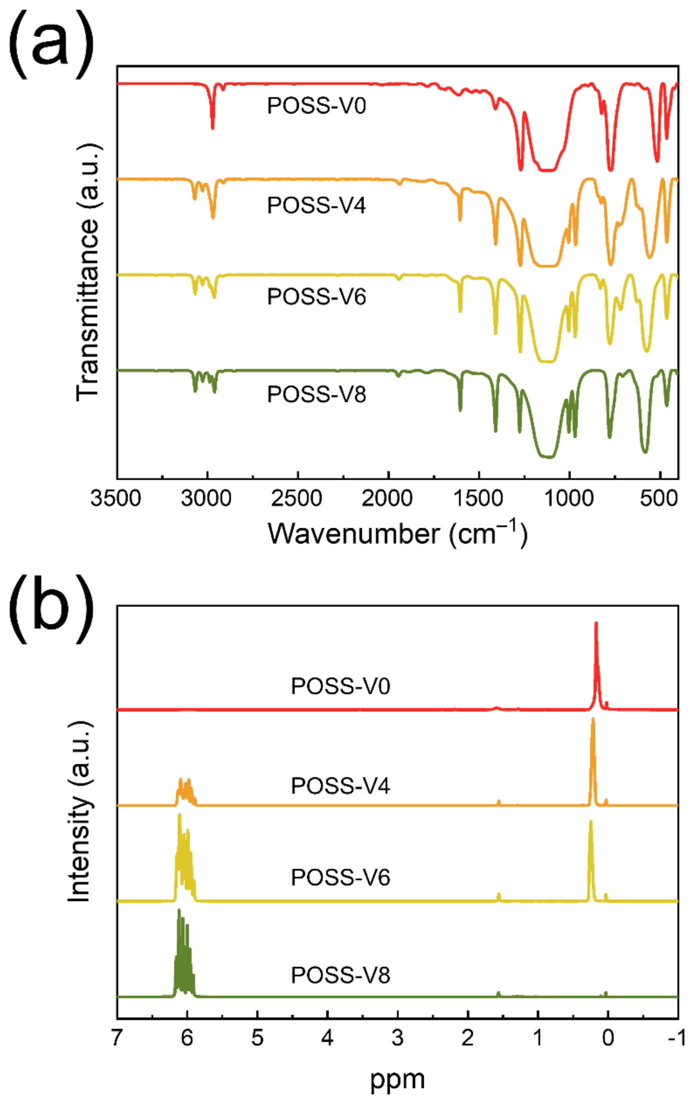

Figure S2. (a) FT-IR spectra of the synthesized POSS-Vs and (b) ^{1}H-NMR spectra of the synthesized POSS-Vs.

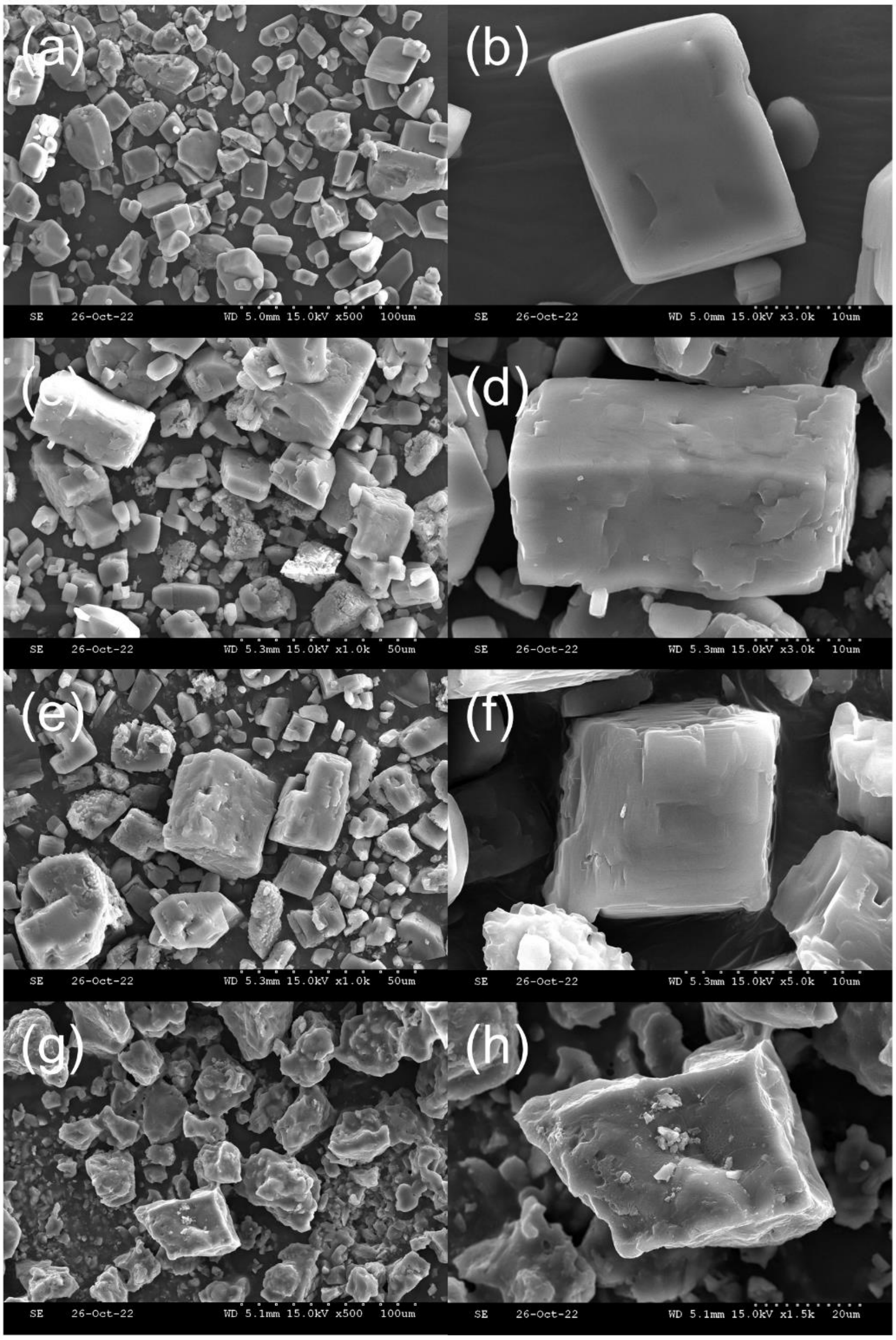

Figure S3. SEM microphotographs of the POSS-Vs: (a), (b) POSS-V8; (c), (d) POSS-V6; (e), (f) POSS-V4; and (g), (h) POSS-V0.

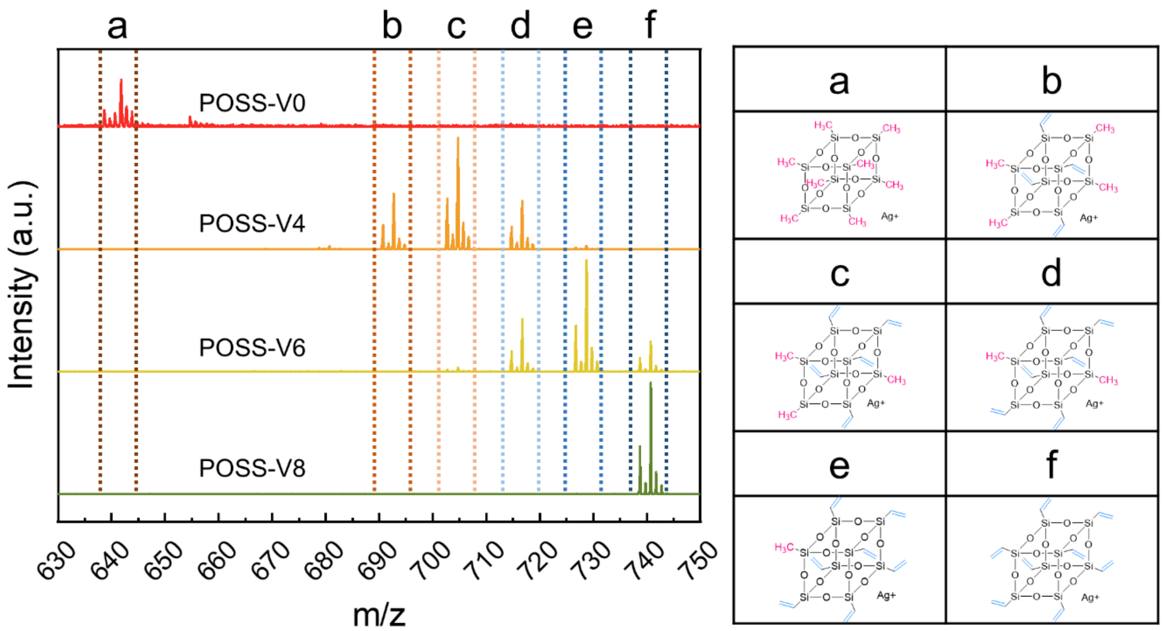

Figure S4. MALDI-ToF MS spectra of all prepared POSS-Vs.
